# Prognostic significance of tumor budding in pancreatic carcinoma: Digitalized image approach evaluation using artificial intelligence.

**DOI:** 10.12688/f1000research.146907.1

**Published:** 2024-04-17

**Authors:** Sarra Ben Rejeb, Jasser Yaacoubi

**Affiliations:** 1 Pathology, Security Forces hospital Tunisia, Tunis, Tunisia

**Keywords:** Pancreatic cancer (PC), tumor budding (TB), prognosis, overall survivor (OS), artificial intelligence.

## Abstract

**Introduction:**

Pancreatic carcinoma (PC) is a highly malignant and lethal tumor characterized by a dismal prognosis which raised the need to identify other prognostic factors for better patient risk stratification. This study investigated the prognostic significance of tumor budding (TB) in pancreatic carcinoma using artificial intelligence.

**Methods:**

In this retrospective multicenter study, we collected all cases of PC diagnosed (2008-2022). TB was assed using 2methods: manuel on hematoxylin-eosin (HE) slides and semi-automated using QUPATH software. The selected slide for each case was digitalized using NIS software version 4.00 connected to the microscope NIKON (Eclipse Ni-U). The pathological images were then incorporated into QUPATH. The budds were counted using cell count functionality based on the nucleus size and pixel variability, and TB scores were categorized as BUDD1(0-4), BUDD2(5-9) and BUDD3(≥10). We analyzed the association between the TB score and prognostic clinicopathological factors and overall survival.

**Results:**

25patients were included (mean age:62.3years;male-to-female ratio:2.57). TB was found in 100%of cases and a high TB score (BUDD2-3) was observed in 56%of cases (using QUPATH versus 48% using HE slides); statistical analysis showed no significant difference between the two methods(p=0.589). A high TB score was associated with older age(>72 years), ductal histological subtype and advanced stage (pT>2).53.8% of patients with lymph node metastasis or advanced stage had high TB score. Multivariate analysis revealed that TB score was strongly and independently associated with overall survival (OS), with a hazard ratio of 2.35.

**Conclusion:**

TB is an additional prognostic factor in PC, and using artificial intelligence via QUPATH software offers a promising and accessible tool for pathologists to evaluate TB and to improve risk stratification in patients with PC.

## Introduction

Pancreatic carcinoma (PC) is an aggressive malignancy with a high rate of recurrence and a 5-year survival rate of <10% even after complete resection and negative lymph nodes status. ^1^ Although tumor resecability and stage remain the most relevant prognostic factors, there is an increasing need to focus on novel histo-prognostic factors that would enable better risk stratification for patients.

Tumor budding (TB), defined as the presence of isolated single cancer cells or clusters of up to four cancer cells at the invasive tumor front, has been recognized as an emerging marker of aggressiveness related to the epithelial to mesenchymal process in patients with colorectal cancer. Thus, TB has been introduced as a routine prognostic marker in colorectal cancer to stratify patients for adjuvant chemotherapy for stage II tumors. ^2^^,^^3^

In PC, although it has been reported that TB has a clear association with adverse prognosis, TB is still not systematically reported by pathologists, and there are no clearly defined recommendations on TB counting in PC.

This study aimed to assess the tumor budding score in PC using artificial intelligence and to explore the association of TB clinicopathological prognostic factors.

## Methods

### Study design

This retrospective, bicentric, cross-sectional study was approved by the Biomedical Research Ethics Committee of our institution (Approval number12/23).

### Patient cohort and clinic-pathological data

We included all cases of pancreatic carcinoma diagnosed in the pathology departments of the Security Forces and Charles Nicolles Hospitals over a period of 14 years (March 2008-December 2022).

We included both biopsies and surgical specimens and excluded patients with other histological subtypes and those for whom large pathological sections were not available. Clinical characteristics of the patients were retrieved from their medical records. Pathological data were collected from pathological reports.

### Tumor budding counting

*Pathological HE slides analysis*

All hematoxylin and eosin (HE)-stained slides were reviewed by two pathologists to select the most representative slides for tumor budding assessment.

Tumor budding was assessed according to the recommendations of the International Tumor Budding Consensus Conference (ITBCC) for colorectal cancer. ^4^ TB was identified as a single tumor cell or a cluster of <5 cells. For each selected HE-stained slide, hotspot areas either at the invasion front or within the tumor center were identified at 10-fold magnification. TB was counted by two pathologists over a field of 0.785 mm ^2^ at 20-fold magnification.

*Digital assessed approach*

**Figure 1.**
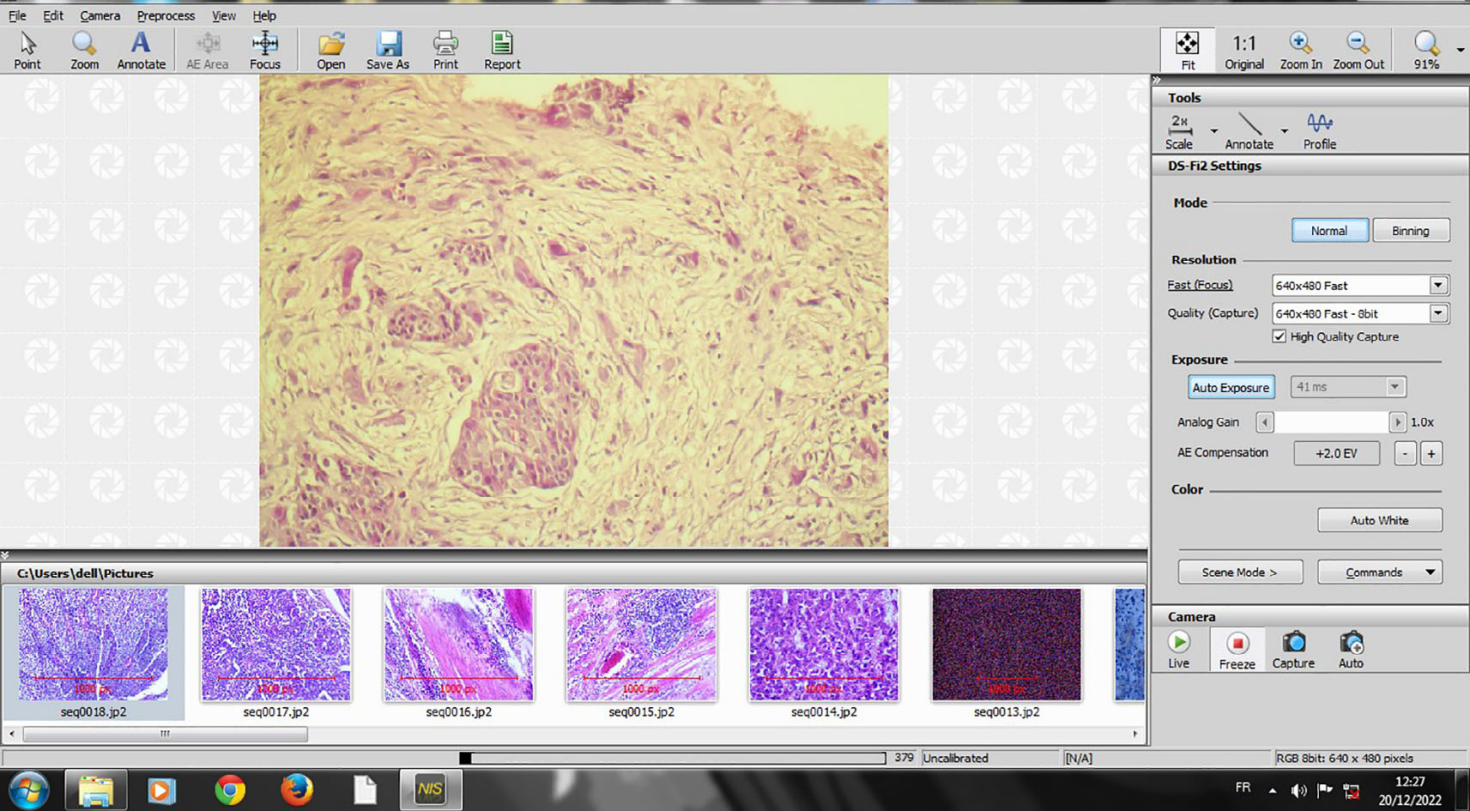
Screenshot of digitalizing an HE X 20 slide on NIS software.

The slides that were selected for TB counting at 20-fold magnification were digitalized using NIS image scope software, which was connected to a Nikon microscope (Eclipse Ni-U) ( Figure 1).

**Figure 2.**
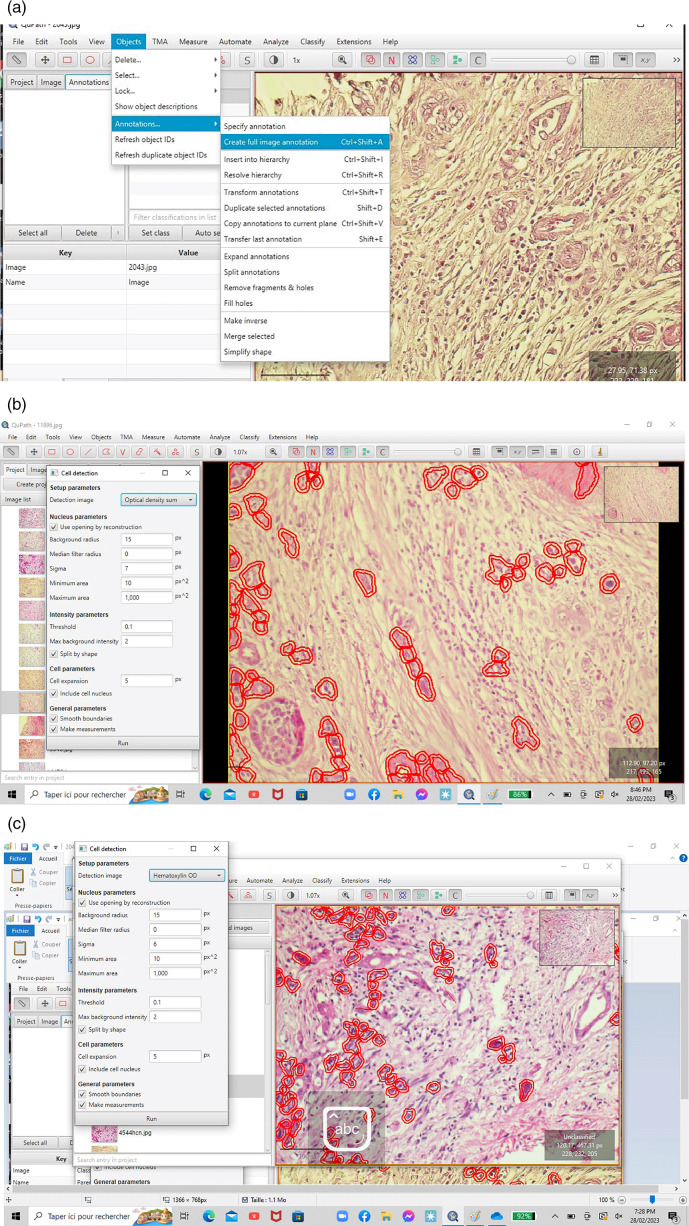
a-b-c: Detection of single tumor cells and small clusters on QUPATH software.

These images were exported in GIF format and then uploaded to open-source software QUPATH (version v0.4.3, https://qupath.github.io/) for digital pathology. ^5^ TB was assessed and evaluated using a semi-automated method according to the methodology of Budeau et al. ^6^ In this study, the author’s assessed TB in a cohort of 92 patients with intrahepatic cholangiocarcinoma. Firstly, Tumor budding was identified in one tissue slide on the basis of the recommendations of the International Tumor Budding Consensus Conference 2016. ^4^ The HE slides were then digitalized and all images were analyzed in QuPath 16 (Version 0.1.2) ( https://qupath.github.io/). Ten rectangles of H&E stained tissue slides were evaluated for both, the tumor-host interface and the tumor center. Each rectangle was standardized for an area of 0.785 mm ^2^ as recommended. The function “cell detection” facilitates the manual differentiation of tumor buds from larger tumor groups by detecting individual cells and only having to assess the number of cells. ^6^ In the present study, according to this methodological approach, we used the annotation functionality of QUPATH to highlight tumor cells (red color). Each 20-fold magnification digitalized slide was segmented into five rectangles, each rectangle corresponded to an area of 0.785 mm ^2^. We used the “cell detection” functionality to circumscribe tumor cells based on nucleus sigma between 3 and 8. The TB score was counted manually for each rectangle, and the final TB score for each case was defined as the average of the five rectangles ( Figure 2a, b and c).

*Classification of tumor budding*

For both morphological and semi-automated methods, the final TB score for each case was categorized into four groups according to the recommendations of ITBCC ^4^:

✓ BD0: no budds
✓ BD1: 1-4 Budds
✓ BD2: 5-10 Budds
✓ BD3:>10Budds

→ The final TB score is calculated as follows:

➢ Low: BD0-BD1
➢ High: BD2-BD3

### Statistical analysis

Statistical analysis was performed using SPSS21 software [(IBM Corp. Released 2021. IBM SPSS Statistics for Windows, Version 28.0. Armonk, NY: IBM Corp) The statistical analyses performed in this article using SPSS21 software can be conducted using the freely accessible software Jamovi https://www.jamovi.org. The user manual is available at the following link. https://lsj.readthedocs.io/ru/latest/Ch03/Ch03_jamoviIntro_1.html. We first used the Kolmogorov-Smirnov test to evaluate the normal distribution and variance homogeneity tests on all continuous variables. Qualitative variables were summarized using frequencies and percentages. Quantitative parameters were summarized using medians and standard deviations. We used Fisher’s exact test to assess the relationship between TB and clinical and pathological parameters. A p-value of less than 5% was considered statistically significant or nearly significant for p [0.05-0.08], considering the small sample size.

## Results

25 cases patients were included in this study. The mean age of the patients was 62.3years old the male-to-female ratio was 2.57. Alcohol consumption and smoking were found in 36% and 56% of the patients, respectively. Clinical signs included abdominal pain (96%), loss of body weight and condition (80%), jaundice (72%), transit disorder (28%), dark urine (64%), discolored stool (44%), pruritus (36%), and fever (16%). On physical examination, a palpable gallbladder was found in 36% and ascites in 12% of patients. Laboratory tests revealed anemia in 64%, elevated CA19.9 in 76%, cytolysis in 52%, and cholestasis in 76%. Surgery was performed in 80% of the cases: cephalic duodeno-pancreatectomy (75%), caudal pancreatectomy (20%), and double bypass (5%). Neoadjuvant therapy was performed in 12% of patients, adjuvant treatment in 56%, and palliative care in 28%. Local recurrence occurred in 44% of patients, distant metastases in 38.9%, and death in 72%. The pathological findings are summarized in Table 1.

**Table 1.** Pathological features of patients.

			Percentage
Histological subtype		**Ductal**	**85%**
		**Intestinal**	10%
		**Adeno-squamous**	5%
Grade		**Low**	72%
		**High**	28%
Peri-neural invasion		**Positive**	80%
		**Negative**	20%
Vascular invasion		**Positive**	**55**%
		**Negative**	45%
Surgical margins		**Positive**	**80%**
		**Negative**	20%
TNM stagging	**T**	**T1**	5%
		**T2**	45%
		**T3**	40%
		**T4**	10%
	**N**	**N0**	45%
		**N1**	45%
		**N2**	10%
	**M**	**M0**	100%

Tumor budding was found in **100%** of cases using the morphological method and **84%** using the digitalized approach. The number varied from 1 to 37 (mean: 8.04, median:4) using the morphological method and from 0 to 19 (mean: 5.92, median: 6) using the QUPATH software. Tumor budding counts are summarized in Table 2.

**Table 2.** Tumor budding counting with both methods.

Budding	Morphological method	Semi-automated method
BUD 1 (0 – 4 buds)	52%(13)	44%(11)
BUD 2 (5 – 9 buds)	16%(4)	44%(11)
BUD 3 (≥10 buds)	32%(8)	12%(3)

A high TB score (BUDD2-3) was found in 48% of the cases using the morphological method and 56% using the semi-automated QUPATH method. No statistically significant differences were observed between the two methods (p=0.589).

### Univariate analysis

Using morphological TB score, a statistically significant association was found between high TB score and advanced age >72 ans (p=0.03). Considering the small sample size, 84.6% of tumors with the ductal subtype had a high TB score, and the difference was nearly statistically significant (p=0.07).

53.8% of patients with lymph node invasion or advanced pT stage had high TB score (p=0.53 and p=0.32).

76.9 Of the patients with perineural invasion, 76.9% had high TB scores. The association between tumor budding and clinicopathological features of pancreatic carcinoma is summarized in Table 3.

**Table 3.** Association of clinic-pathological characteristics with tumor budding.

	Low TB (n=12)	High TB (n=13)	p
Age >72 ans	0	4	**0.03**
	0%	30.7%	
Male	10	8	0.22
	83.3%	61.5%	
Smoking	9	5	0.07
	75%	35.7%	
Ascitis	0	3	0.09
	0%	23%	
High tumor size	9	8	0.38
	75%	61.5%	
Subtype			
Ductal	6	11	**0.07**
	50%	**84.6%**	
Intestinal	2	0	0.22
	16.6%	0.0%	
Adenosquamous	0	1	0.52
	0.0%	7.6%	
Surgical margins	1	3	0.46
	8.3%	23%	
Peri-neural invasion	6	10	0.53
	50%	**76.9%**	
Vascular invasion	3	8	0.20
	25%	**61.5%**	
High Grade	1	4	0.61
	8.3%	30.7%	
T >2	6	4	**0.08**
	50%	30.7%	
N+	4	7	0.53
	25%	**53.8%**	
M+	6	5	0.56
	50%	38.4%	
Advanced stage	7	7	0.32
	58.3%	**53.8%**	

### Multivariate analysis

**Figure 3.**
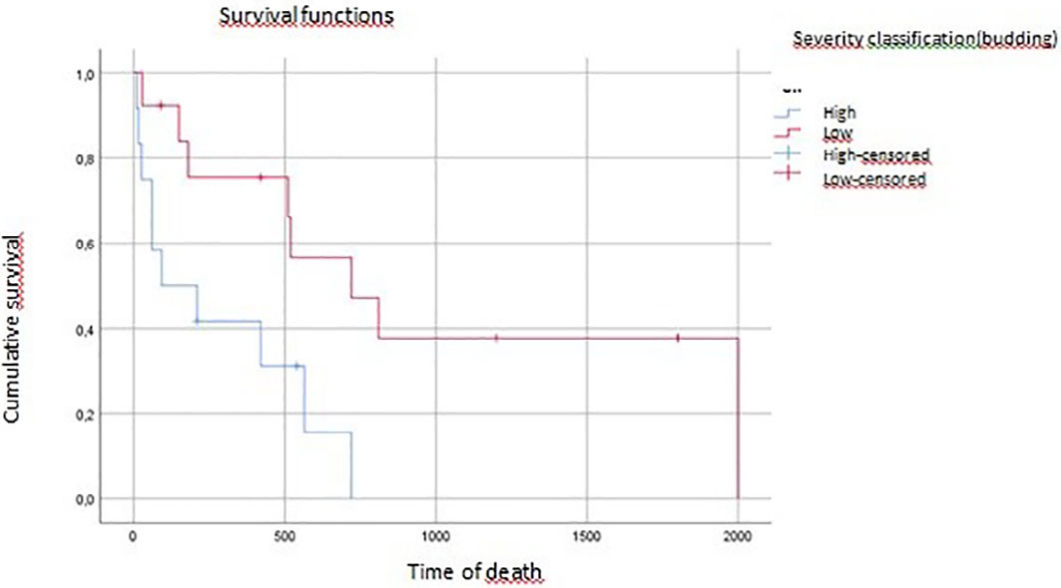
Tumoral budding impact on overall survival (time of death).

**Figure 4.**
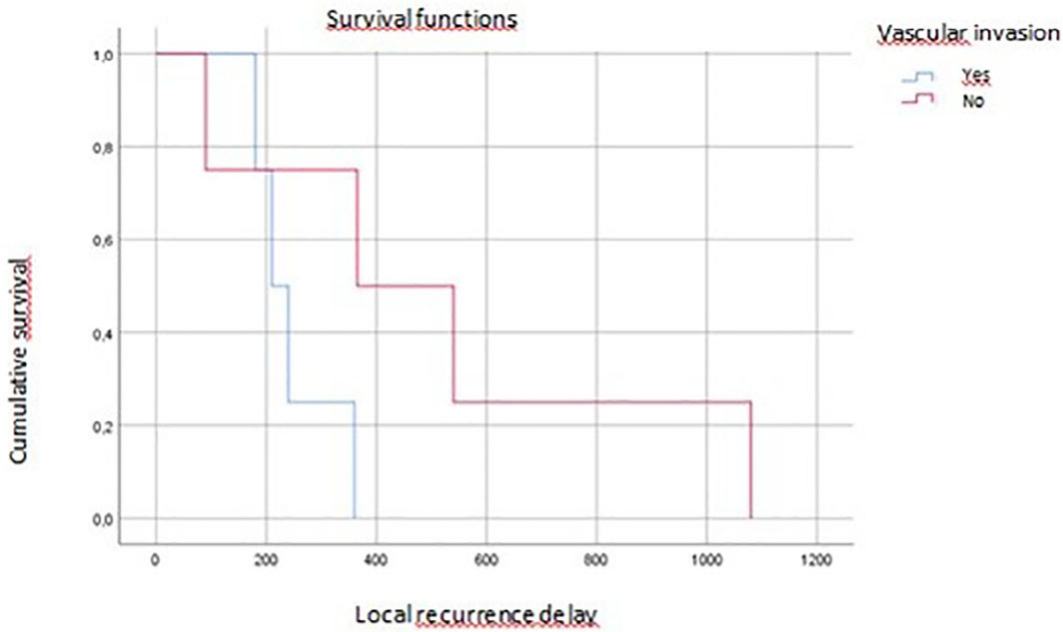
Vascular invasion impact on local recurrence.

**Figure 5.**
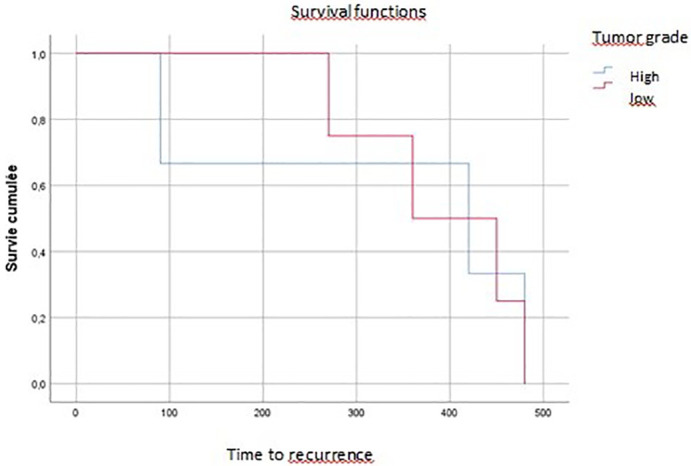
Tumor grade impact on recurrence.

On multivariate analysis, tumor grade, vascular invasion, and tumor budding affected overall survival (p=0.04, p=0.07, p=0.016, respectively) ( Figures 3, 4, 5).

## Discussion

In the present study, tumor budding was found in 100%of cases using morphological methods and 84% of cases using the digitalized approach; these findings are consistent with previous reports indicating that TB is reported in approximately 85-100% of specimens with pancreatic carcinoma. ^8^^–^^10^ This fact raises the hypothesis that TB is a relatively constant finding that could also be a pervasive feature of pancreatic carcinoma, especially in biopsy specimens. ^10^

In our study, a high TB score was found in 48% (VS 56% using QUPATH), which is slightly lower than that reported in previous studies reporting a high TB score in about 56 to 80% of cases. ^8^^–^^10^ These disparities may be partly explained by the differences in TB counting methods, the surface of the HPF, and the use of immunohistochemistry in some studies to identify CK+-stained tumor cells. TB is defined as single cells or clusters of <5 cells at the tumor invasion front. ^2^^,^^4^ This quantifiable histological feature has gained attention over the last 10years and has proven its prognostic value in many cancers. The International Tumor Budding Consensus Conference (ITBCC) proposed a scoring system for tumor buds in colorectal carcinomas that was later applied to other cancers such as hepatocellular, oral squamous cell, and bladder cancers. ^4^^,^^6^ However, there is still no standardized method for reporting tumor budding in PC, and various approaches are available to count tumor buds either on HE slides or using pathological image analysis softwares. ^6^^,^^11^ In the present study, we first assessed tumor budding in pancreatic carcinoma specimens according to the recommendations of the ITBCC at 20-fold magnification for a field of 0.785 mm ^2^, either at the tumor front or within the tumor center. Then, similar to the published study of Budeau et al., ^6^ we extrapolated the ITBCC counting approach on an optic microscope to a digital image analysis system using QUPATH, an open-access software for digital image analysis. QUPATH is completely free, which enables pathologists in low-income countries to have access to digital pathology analysis. ^5^ The tumor cell detection functionality of the QUPATH software facilitates distinguishing between tumor cells and small clusters from a larger group of cells. In addition, compared to “analog” microscopy, QUAPTH offers the possibility of re-evaluating tumor buds at anytimes higher than those of other pathologists. ^6^ Although a high TB score was found in 48% of patients using the first method compared with 56% using the digitalized approach, the difference was not statistically significant (p=0.589). Hence, QUPATH could be an interesting, costless, and accurate alternative for pathologists, which would considerably reduce the work time and facilitate TB counting.

According to the recommendations of ITBCC, the TB score is categorized as a three-tier grading system for patient risk stratification. ^4^^,^^6^ In the present study, similar to the study published by Tanaka et al., ^12^ we first graded the TB score as low (BUDD1), intermediate (BUDD2), and high (BUDD3) but later combined BUDD2 and BUDD3 groups into high TB scores for statistical analysis purpose. Consequently, we compared the high and low TB score groups using clinicopathological and survival data.

Using the morphological method results, we demonstrated a statistically significant (or nearly significant) association between high tumor budding and advanced age >72 years (p=0.03), ductal subtype (p=0.07), and advanced-stage pT>pT2 (p=0.08). These results are consistent with those of previous studies that demonstrated an association between high TB and prognostic factors. In a study published by O’Connor et al., ^9^ high-grade budding (> 10 buds in 10 HPFs) was associated with a high tumor grade, lymphovascular invasion, and perineural invasion. In our study, 53.8% of patients with lymph node invasion or advanced stage had a high TB score; likewise, 76.9% of patients with perineural invasion had a high TB score. However, these findings were not statistically significant (p=0.53, p=0.32, and p=0.53, respectively), which could be related to the small sample size in our study. Of note, regardless of the method used, most published studies on pancreatic cancer did not reveal a statistically significant association between TB and prognostic factors. ^8^^,^^10^^,^^13^^–^^15^

In the present study, the authors described a statistically significant association between tumor budding and overall survival (p=0.016) in the multivariate analysis after adjusting for other significant variables. This finding is consistent with previous reports that support the prognostic value of the TB score in pancreatic carcinoma for better patient stratification and treatment guiding. ^8^^–^^11^ Although these results are promising, there are still many discrepancies in the TB counting methods. First, some authors only considered TB at the tumor-host interface ^8^; however, there is increasing evidence that intra-tumor and peri-tumor buds have comparable prognostic significance. ^4^^,^^6^^,^^11^^–^^17^ Second, some authors suggested that tumor budding evaluation is more accurate and reproducible using immunohistochemistry with anti-CK antibody ^18^; however, in a multicenter study, Hacking S et al. ^19^ demonstrated that although immunohistochemical staining facilitates the detection of tumor cells, it has comparable intra- and inter-observatory reproducibility to the HE slides TB counting approach. Finally, in the era of artificial intelligence, using digital pathology for TB assessment is an interesting alternative approach that could considerably reduce the examination time and offer better accuracy and reproducibility. In this context, many studies have demonstrated a high range of diagnostic concordance (90-99%) between digital slides and conventional glass slides. ^20^^,^^21^ Many digital pathology image software packages have been developed; however, access to these platforms remains difficult for some pathologists. ^22^^,^^23^ Hence, QUPATH software could be an interesting alternative for integrating digital pathology in routine practice. A consensus on tumor budding counting and reporting on digitalized slides in pancreatic carcinoma is necessary to definitively integrate TB in pathology reports.

## Conclusions

Our results demonstrated that tumor budding either assessed manually on HE slides or semi-automated on digitalized images is a relatively constant finding in pancreatic carcinoma, with a high score in about 50% of cases. Our findings also showed a high concordance of both methods in TB assessment, supporting the benefits of integrating digital pathology in routine practice. Finally, our results provide further evidence for the potential prognostic value of TB in pancreatic carcinoma. However, our study has some limitations, such as its retrospective design and small sample size. Further studies on TB counting using QUAPATH in pancreatic carcinoma are needed to integrate this method into routine practice for better risk stratification.

## Ethics approval

This research was conducted following the ethical guidelines outlined by the Ethics committee of the Internal Security Forces Hospital (Obtained on 1 ^st^ December 2023, approval number 12/23). All procedures involving human tissues were approved by the committee and were performed in accordance with the ethical standards laid down in the 1964 Declaration of Helsinki and its later amendments as well as the National Medical Code of Ethics (Title VI, Article 99 to 111). Verbal informed consent was obtained at admission from all individual participants included in the study. The majority of patients were illiterate, unable to read or write, therefore verbal consent was preferred. Confidentiality and anonymity of participants were strictly maintained throughout the study. Any potential conflicts of interest have been disclosed and managed appropriately. Confidentiality and anonymity of participants were strictly maintained throughout the study. Any potential conflicts of interest have been disclosed and managed appropriately.

Link to National Medical Code of Ethics: chrome-extension://efaidnbmnnnibpcajpcglclefindmkaj/ http://www.atds.org.tn/Decretdeontologiemedicale93.pdf

## Authors contribution

Sarra Ben Rejeb: Conceptualization Investigation; Methodology Writing Validation;

Jasser yaacoubi:; Data curation; Formal analysis;

## Data Availability

Figshare: Tumor budding in pancreatic carcinoma. https://doi.org/10.6084/m9.figshare.25265755.v2 Data are available under the terms of the Creative Commons Attribution 4.0 International license (CC-BY 4.0). figshare: STROBE Checklist for Tumor budding in pancreatic carcinoma https://doi.org/10.6084/m9.figshare.25265755.v2 Data are available under the terms of the Creative Commons Attribution 4.0 International license (CC-BY 4.0). Software availability statement: The statistical analyses performed in this article using SPSS21 software can be conducted using the freely accessible software Jamovi https://www.jamovi.org Source code available from: https://github.com/jamovi/jamovi/tree/v2.2.4 Archived software available from: [DOI specific to version 2.2.4, typically accessible from Zenodo] License: OSI approved open license software is under GPL-3.0 License The user manual is available at the following link. https://lsj.readthedocs.io/ru/latest/Ch03/Ch03_jamoviIntro_1.html
