# Supplementary material for: Prognostic significance of tumor budding in pancreatic carcinoma: Digitalized image approach evaluation using artificial intelligence
Source: F1000Res. 2025 Sep 2;13:282. Originally published 2024 Apr 17. [Version 3] doi: 10.12688/f1000research.146907.3 (PMC12519071; doi:10.12688/f1000research.146907.3)
Supplement: Peer review history

# Reviewer report: Reviewer response for version 3

**DOI:** 10.5256/f1000research.187231.r411215

**Published:** 2025-10-13

**Authors:** Bipneet Singh

**Affiliations:** 1 University of Kentucky (Ringgold ID: 4530), Lexington, Kentucky, USA; 2 Henry Ford Health System, Detroit, Michigan, USA

Authors have made modifications per the feedback. In the current status, I am comfortable with the indexing.

Is the work clearly and accurately presented and does it cite the current literature?

Yes

If applicable, is the statistical analysis and its interpretation appropriate?

I cannot comment. A qualified statistician is required.

Are all the source data underlying the results available to ensure full reproducibility?

Yes

Is the study design appropriate and is the work technically sound?

Partly

Are the conclusions drawn adequately supported by the results?

Partly

Are sufficient details of methods and analysis provided to allow replication by others?

Yes

Reviewer Expertise:

Gastroenterology, Hepatology, Oncology

I confirm that I have read this submission and believe that I have an appropriate level of expertise to confirm that it is of an acceptable scientific standard.

# Reviewer report: Reviewer response for version 2

**DOI:** 10.5256/f1000research.176229.r399399

**Published:** 2025-08-23

**Authors:** Bipneet Singh

**Affiliations:** 1 University of Kentucky (Ringgold ID: 4530), Lexington, Kentucky, USA; 2 Henry Ford Health System, Detroit, Michigan, USA

1. Give a line about tumor budding in the abstract introduction
2. “Manuel” is misspelled
3. “25patients”, there has to be a space here
4. “Pancreatic carcinoma (PC) is an aggressive malignancy with a high rate of recurrence and a 5-year survival rate of <10% even after complete resection and negative lymph nodes status.” Kindly restructure the sentence. The sentence can be split into 2-3 parts, explaining the staging, progression, treatment, and relapse rates.
5. “Although tumor resectability and stage remain the most relevant prognostic factors, there is an increasing need to focus on novel histo-prognostic factors that would enable better risk stratification for patients.” Take “need of” out of the sentence.
6. “resecability” is spelled wrong.
7. “In PC, although it has been reported that TB has a clear association with adverse prognosis.” This requires a citation.
8. The introduction needs to be expanded.
9. “We included in this study all patients with primary pancreatic adenocarcinoma diagnosed on surgical specimens or pancreatic biopsies in the pathology departments of the Security Forces and Charles Nicolles Hospitals over a period of 14 years (March 2008-December 2022).” Remove “in this study”
10. Why are there two separate non-inclusion and exclusion criteria?
11. “author’s” to be replaced with “authors”
12. “ In the present study, we first assessed tumor budding in pancreatic carcinoma specimens according to the recommendations of the ITBCC at 20-fold magnification for a field of 0.785 mm ^2^, either at the tumor front or within the tumor center. Then, similar to the published study of Budeau et al., ^6^ we extrapolated the ITBCC counting approach on an optic microscope to a digital image analysis system using QUPATH, an open-access software for digital image analysis. QUPATH is completely free, which enables pathologists in low-income countries to have access to digital pathology analysis. ^5^ The tumor cell detection functionality of the QUPATH software facilitates distinguishing between tumor cells and small clusters from a larger group of cells. In addition, compared to “analog” microscopy, QUAPTH offers the possibility of re-evaluating tumor buds at anytime higher than those of other pathologists. ^6^ Although a high TB score was found in 48% of patients using the first method compared with 56% using the digitalized approach, the difference was not statistically significant (p=0.589). Hence, QUPATH could be an interesting, costless, and accurate alternative for pathologists, which would considerably reduce the work time and facilitate TB counting.” Relevance?
13. Expand on the relation between TB and prognosis in the discussion.

Is the work clearly and accurately presented and does it cite the current literature?

Yes

If applicable, is the statistical analysis and its interpretation appropriate?

I cannot comment. A qualified statistician is required.

Are all the source data underlying the results available to ensure full reproducibility?

Yes

Is the study design appropriate and is the work technically sound?

Partly

Are the conclusions drawn adequately supported by the results?

Partly

Are sufficient details of methods and analysis provided to allow replication by others?

Yes

Reviewer Expertise:

Gastroenterology, Hepatology, Oncology

I confirm that I have read this submission and believe that I have an appropriate level of expertise to confirm that it is of an acceptable scientific standard, however I have significant reservations, as outlined above.

# Response

**Published:** 2025-08-25

**Authors:** Sarra Ben Rejeb

**Affiliations:** Pathology, Universite de Tunis, Tunis, Tunis, Tunisia

**Dear Reviewer, we greatly appreciate the valuable feedback you provided which has significantly improved the clarity, depth, and scientific rigor of our work.**

**In response to the your comments, we have made the following revisions:**

1. **Added: "Tumor budding (TB), defined as isolated single cancer cells or small clusters of up to four cells at the invasive front, is an emerging histoprognostic factor associated with aggressiveness in various malignancies."**
2. **Corrected to "manual" in the Methods section.**
3. **Corrected to "25 patients" in the Results section.**
4. **“Pancreatic carcinoma (PC) is an aggressive malignancy with a high rate of recurrence and a 5-year survival rate of <10% even after complete resection and negative lymph nodes status.” Kindly restructure the sentence. The sentence can be split into 2-3 parts, explaining the staging, progression, treatment, and relapse rates.: ⇒ We restructured and expanded with updated data.**
5. **“Although tumor resectability and stage remain the most relevant prognostic factors, there is an increasing need to focus on novel histo-prognostic factors that would enable better risk stratification for patients.” Take “need of” out of the sentence.: Corrected to "there is an increasing need to focus".**
6. **Resecability is spelled wrong.: Corrected to "resectability".**
7. **“In PC, although it has been reported that TB has a clear association with adverse prognosis.” This requires a citation.: Added citations from recent studies.**
8. **The introduction needs to be expanded.: Expanded with epidemiology, TB in other cancers, biological mechanisms (e.g., EMT), and specific studies on TB in PC.**
9. **We included in this study all patients with primary pancreatic adenocarcinoma diagnosed on surgical specimens or pancreatic biopsies in the pathology departments of the Security Forces and Charles Nicolles Hospitals over a period of 14 years (March 2008-December 2022).” Remove “in this study”: Removed "in this study".**
10. **Why are there two separate non-inclusion and exclusion criteria? ⇒ We Combined into a single "Exclusion Criteria" section for clarity.**
11. **“author’s” to be replaced with “authors”: Corrected to "authors".**
12. **The paragraph on QUPATH methods and relevance?: Retained with added relevance statement.**
13. **Expand on the relation between TB and prognosis in the discussion.: Further expanded with evidence from recent meta-analyses and studies (e.g., 2019-2024), including specific associations with OS, DFS, HRs (e.g., pooled HR 2.13 for OS), links to EMT, neoadjuvant therapy contexts, and recommendations for routine reporting and risk stratification. Incorporated new citations from literature search.**

# Reviewer report: Reviewer response for version 2

**DOI:** 10.5256/f1000research.176229.r358161

**Published:** 2025-01-21

**Authors:** Ozden Oz

**Affiliations:** 1 Izmir Bozyaka Training and Research Hospital, University of Health Sciences, Izmir, Turkey

The explanations made on the suggestions I made are sufficient.

 My best 

 M.D. Ozden oz

Is the work clearly and accurately presented and does it cite the current literature?

Partly

If applicable, is the statistical analysis and its interpretation appropriate?

Yes

Are all the source data underlying the results available to ensure full reproducibility?

Yes

Is the study design appropriate and is the work technically sound?

Yes

Are the conclusions drawn adequately supported by the results?

Yes

Are sufficient details of methods and analysis provided to allow replication by others?

Yes

Reviewer Expertise:

clinic pathology

I confirm that I have read this submission and believe that I have an appropriate level of expertise to confirm that it is of an acceptable scientific standard.

# Reviewer report: Reviewer response for version 1

**DOI:** 10.5256/f1000research.161038.r305079

**Published:** 2024-08-09

**Authors:** Ozden Oz

**Affiliations:** 1 Izmir Bozyaka Training and Research Hospital, University of Health Sciences, Izmir, Turkey

First of all, it is a well-designed study and seems applicable.

 Major

 1. The criteria by which patients were selected should be written in more detail. 

 None of the patients appear to have metastases (table 1). Was a selection made in this regard?

 2. It should be discussed why tumor budding could not be found in 16% of the patients with QUPATH method.

 3. It should be discussed why a higher TB degree 3 was found with the morphological method.

 5. Figure 3.4.5 Univariate analysis (Kaplan-Meier) ? or multivariate Cox?  It must be clear

 Minor 

 1. Details of tumor budding in pancreatic cancer should be more supported by literature. 

 Such as:

 DOI: 10.4103/ijpm.ijpm_905_21 etc.

Is the work clearly and accurately presented and does it cite the current literature?

Partly

If applicable, is the statistical analysis and its interpretation appropriate?

Yes

Are all the source data underlying the results available to ensure full reproducibility?

Yes

Is the study design appropriate and is the work technically sound?

Yes

Are the conclusions drawn adequately supported by the results?

Yes

Are sufficient details of methods and analysis provided to allow replication by others?

Yes

Reviewer Expertise:

clinic pathology

I confirm that I have read this submission and believe that I have an appropriate level of expertise to confirm that it is of an acceptable scientific standard, however I have significant reservations, as outlined above.

## References

[1] : Evaluation and prognostic significance of tumor budding in pancreatic ductal adenocarcinomas. *Indian J Pathol Microbiol* .2023;66(1) : 10.4103/ijpm.ijpm_905_21 38-43 10.4103/ijpm.ijpm_905_21 36656208

[2] : The Significance of Tumor Budding and Immunohistochemical Axl Expression in Gallbladder Adenocarcinomas. *Balkan Med J* .2022;39(3) : 10.4274/balkanmedj.galenos.2022.2021-9-37 199-208 10.4274/balkanmedj.galenos.2022.2021-9-37 35430785 PMC9136546

# Response

**Published:** 2024-11-06

**Authors:** Sarra Ben Rejeb

**Affiliations:** Pathology, Universite de Tunis, Tunis, Tunis, Tunisia

Response to Reviewer 1: 

 Dear Reviewer, thank you for your valuable  comments. 

**1- Regarding our inclusion criteria:** we included all cases of primary pancreatic adenocarcinoma diagnosed on surgical specimens or biopsies in the pathology departments of the security forces hospital and Charles Nicolles Hospital, regardless of the stage of the disease, whether metastatic or localized. However, at the time of data collection, none of our patients had known metastases at diagnosis by mere coincidence. This was not a selection criterion. It is worth noting, however, that during follow-up, 20% of the patients developed metastases .

**2- To further clarify our inclusion criteria:**

**2.1. Inclusion Criteria:**

 We included in this study all patients with primary pancreatic adenocarcinoma diagnosed on surgical specimens or pancreatic biopsies in the Department of Pathology and Cytology at FSI Hospital and HCN during the study period.

**2.2. Non-inclusion Criteria:**

 The following cases were not included in this study:

- Patients with intraductal papillary mucinous tumors without invasive foci.
- Other histological types (neuroendocrine or mesenchymal tumors).
- Patients with ampullary, gallbladder, or bile duct adenocarcinoma.

**2.3. Exclusion Criteria:**

 We excluded from our study:

- Patients with unusable clinical records.
- Small and non-representative biopsy samples.
- Biopsy samples of suboptimal technical quality that created artifacts during image digitization

**3-  Regarding tumor budding using QUPATH:**

 Through our study, tumor budding was observed in 100% of cases by morphological analysis and in 84% with the QUPATH software. This suggests that using a semi-automated approach may offer greater analytical precision by reducing false positives. These cases likely corresponded to atypical mesenchymal fibroblastic or myofibroblastic cells or poorly differentiated tumor glands that were mistaken for isolated tumor cells

**4- High TB on morphological method :**

 Similarly, using the QUPATH software reduced the number of cases classified as high BUDD3 with the morphological approach. This is due to the software's precision in distinguishing between isolated tumor cells or clusters of fewer than 5 cells, which meet the definition of tumor budding, as opposed to poorly differentiated carcinoma clusters or remnants of neuroendocrine islets that were misdiagnosed as TB on HE slides examination and were  sofalse positives. Indeed, with its annotation features, the QUPATH software allows for the identification and deselection of false positives

**5- Survival curves :** Survival data were analyzed by generating survival curves using the Kaplan-Meier method and compared using the Log-rank test in univariate analysis

***Minor Revisions:***

 1- This publication was not found during the literature search and manuscript writing, but it is highly relevant to our research topic.
